# Nociceptive thermal threshold testing in horses – effect of neuroleptic sedation and neuroleptanalgesia at different stimulation sites

**DOI:** 10.1186/1746-6148-9-135

**Published:** 2013-07-09

**Authors:** Christin Poller, Klaus Hopster, Karl Rohn, Sabine BR Kästner

**Affiliations:** 1 Clinic for Horses, University of Veterinary Medicine Hannover Foundation, Hannover, Germany; 2 Department of Biometry, Epidemiology and Information Processing, University of Veterinary Medicine Hannover Foundation, Hannover, Germany; 3 Clinic for Small Animals, University of Veterinary Medicine Hannover Foundation, Hannover, Germany

**Keywords:** Horse, Nociception, Contact heat, Buprenorphine, Different body sites

## Abstract

**Background:**

Aim of the study was to compare the effect of neuroleptic sedation with acepromazine and neuroleptanalgesia with acepromazine and buprenorphine on thermal thresholds (TT) obtained at the nostrils and at the withers. The study was carried out as a randomized, blinded, controlled trial with cross-over design. Thermal thresholds were determined by incremental contact heat applied to the skin above the nostril (N) or the withers (W). Eleven horses were treated with saline (S), acepromazine (0.05 mg/kg) (ACE) or acepromazine and buprenorphine (0.0075 mg/kg) (AB) intravenously (IV). Single stimulations were performed 15 minutes prior and 15, 45, 75, 105, 165, 225, 285, 405 and 525 minutes after treatment. Sedation score, gastrointestinal auscultation score and occurrence of skin lesions were recorded. Data were analysed with analysis of variance for repeated measurements.

**Results:**

There were no significant differences in TT between N and W with all treatments. The TT remained constant after S and there was no difference in TT between S and ACE. After AB there was a significant increase above baseline in TT until 405 minutes after treatment. Restlessness occurred 30–90 minutes after AB in 7 horses. All horses had reduced to absent borborygmi after AB administration for 165 to 495 minutes.

**Conclusion:**

Thermal stimulation at both described body areas gives comparable results in the assessment of cutaneous anti-nociception in horses. There is no differential influence of neuroleptic sedation or neuroleptanalgesia on TTs obtained at N or W. Buprenorphine combined with acepromazine has a long lasting anti-nociceptive effect associated with the typical opioid induced side effects in horses.

## Background

Thermal stimulation is a natural modality to induce nociception. In addition, it is able to stimulate C-fibre nociceptors and is therefore suitable to detect opioid induced antinociception [1,2]. In horses, radiant heat stimulating the lateral aspect of the fetlock, the coronary band and the withers was used to determine the latency of the hoof withdrawal reflex (HWR) or skin twitching reflex (STR) after fentanyl [3], local anaesthetics [4] or buprenorphine [5]. Contact heat thermal threshold testing by ramped heating has been used for the detection of the analgesic efficacy of lidocaine [6], fentanyl and butorphanol [7,8] and buprenorphine [9] in horses by performing thermal stimulation at the withers.

However, the type of nocifensive response and its detection varies between body regions and might therefore influence the determination of thermal thresholds. Transmission of a noxious stimulus at the body travels through the dorsal horn of the spinal cord and ascends to the brain via the spinothalamic tract [10]. Assessment of the skin twitch in response to nociceptive stimulation at the withers involves direct reflex pathways (cutaneous trunci reflex). In contrast, sensory innervation of the forehead is carried by the trigeminal nerve and the trigeminal ganglion entering the brainstem at the level of the pons [10]. Nocifensive reflexes in response to painful stimulation of the tooth root (“jaw opening reflex”) or the supraorbital nerve (trigeminocervical reflex) with contraction of the splenius muscle (head jerking) are described in different species [11-13]. However, coordinated behaviour like head shaking or rubbing the face against an object can be considered as result of conscious perception. Nociceptive stimulation at the head or the body might result in differences in thermal thresholds, depending on recognition and definition of end points. In addition, drugs affecting alertness might have differential influences on reflex responses and conscious reactions.

Therefore, the aim of the study was to compare the effect of neuroleptic sedation with acepromazine and neuroleptanalgesia with acepromazine and buprenorphine on TTs obtained at the nostrils and at the withers. The hypothesis was that neuroleptic sedation and neuroleptanalgesia would inhibit conscious reactions to a greater extent than reflex responses.

## Results

### Skin temperature

There was no significant change in skin temperature at N over the course of the experiment in group S or ACE, but in group AB there was a statistically significant increase in skin temperature over the first measurements (Table 1). Skin temperatures at the withers increased significantly in group S and AB for the first measurements and decreased significantly in group ACE 165 minutes after injection for the next four hours (Table 1).

**Table 1:** **Mean** (± **SD**) **skin temperatures in 11 horses at the withers or nostrils**

**Medication**	**Saline solution**		**Acepromazine (0.05 mg/kg)**		**Acepromazine (0.05 mg/kg)/Buprenorphine (0.0075 mg/kg)**	
**Location/ Time (min)**	**Nostril (°C)**	**Withers (°C)**	**Nostril (°C)**	**Withers (°C)**	**Nostril (°C)**	**Withers (°C)**
Baseline	31.8 ± 2.2^a^	31.2 ± 2.5^a^	31.1 ± 2.6^a^	32.1 ± 1.7^a^	31.7 ± 1.6^a^	30.8 ± 2.4^a^
15	32.8 ± 1.1^a^	32.8 ± 1.3^b^	31.9 ± 1.5^a^	32.9 ± 1.4^a^	32.9 ± 1.6^b^	33.6 ± 2.1^b^
45	32.7 ± 0.5^a^	32.9 ± 1.2^b^	32.9 ± 0.9^b^	32.8 ± 1.6^a^	33.2 ± 1.2^b^	33.1 ± 1.2^b^
75	32.6 ± 1.0^a^	32.7 ± 1.0^b^	32.3 ± 1.6^a^	32.4 ± 1.9^a^	33.2 ± 1.2^b^	33.1 ± 1.0^b^
105	32.9 ± 0.7^a^	33.2 ± 1.0^b^	31.8 ± 1.7^a^	32.1 ± 1.5^a^	32.5 ± 1.2^a^	32.8 ± 1.1^b^
165	32.1 ± 0.9^a^	31.1 ± 1.4^a^	31.1 ± 1.4^a^	30.1 ± 2.1^b^	32.4 ± 1.7^a^	31.1 ± 2.6^a^
225	31.9 ± 1.0^a^	31.1 ± 1.1^a^	30.9 ± 1.7^a^	29.7 ± 1.8^b^	32.2 ± 1.3^a^	31.4 ± 2.1^a^
285	31.4 ± 1.0^a^	31.7 ± 1.3^a^	31.4 ± 1.2^a^	30.0 ± 2.0^b^	32.5 ± 1.1^a^	31.9 ± 2.1^a^
405	31.7 ± 0.9^a^	31.1 ± 1.5^a^	31.6 ± 1.5^a^	30.3 ± 1.8^b^	32.4 ± 0.9^a^	31.4 ± 2.0^a^
525	31.8 ± 1.0^a^	31.5 ± 1.6^a^	31.6 ± 1.8^a^	31.5 ± 2.1^a^	32.5 ± 1.8^a^	32.2 ± 2.1^b^

Mean (± SD) skin temperatures in 11 horses at the withers or nostrils treated with saline, acepromazine or acepromazine + buprenorphine intravenously. Fifteen minutes before drug administration baseline measurement was performed and the first measurement was started 15 minutes after drug injection.

^a, b^ = values with different superscripts differ significantly within each treatment group (*p* < 0.05).

### Thermal threshold

**Figure 1:**
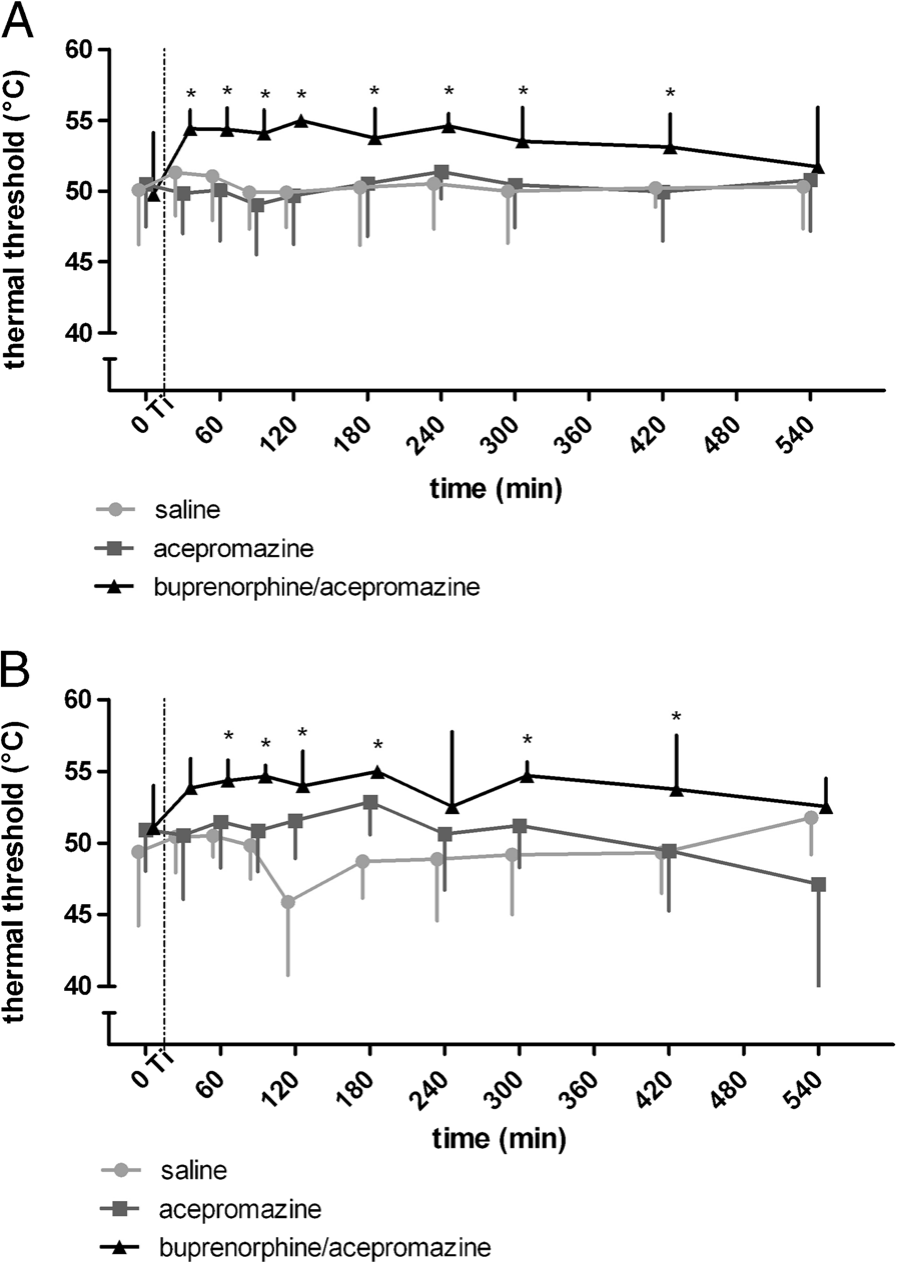
**Mean (± SD) thermal threshold temperature (°C) in 11 horses at the withers or nostrils.** Mean (± SD) thermal threshold temperature (°C) in 11 horses at the withers or nostrils treated with saline, acepromazine or acepromazine + buprenorphine 0: baseline measurement; Ti: time point of injection (15 min); *: significant (p < 0.05) difference in thermal threshold compared to baseline **A**: thermal threshold temperatures at the nostril **B**: thermal threshold temperatures at the withers.

**Figure 2:**
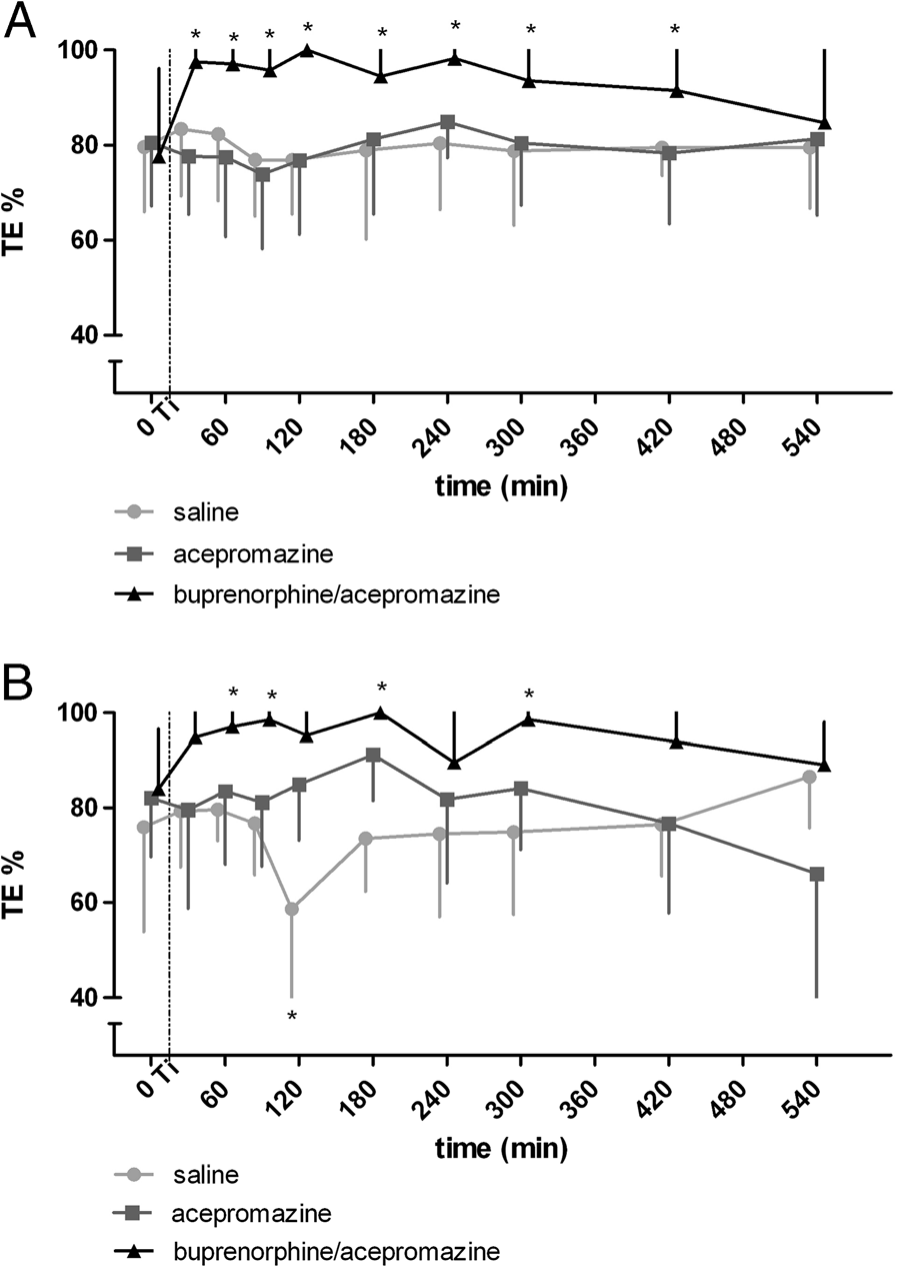
**TE (%) in 11 horses at the withers or nostrils.** TE (%) in 11 horses at the withers or nostrils treated with saline, acepromazine or acepromazine + buprenorphine TE  %   =  100 × ([T_T_ ‒ T_0_]/[T_c_ ‒ T_0_]). T_T_ is the thermal threshold temperature, T_0_ is the skin temperature and T_c_ is the thermal cut-out temperature. 0: baseline measurement; Ti: time point of injection (15 min); *: significant (p < 0.05) difference in thermal threshold compared to baseline **A**: TE (%) at the nostril **B**: TE (%) at the withers.

Thermal threshold temperatures at the nostril did not change over the observation period in group S and group ACE (Figure 1A). In group AB, TTs were significantly increased from 15 minutes until 405 minutes after treatment compared to the baseline measurement (Figure 1A) and compared to TTs in group S and group ACE. At W, there was also no change in TTs after treatment in group S and group ACE (Figure 1B). Similar to stimulation at N TTs increased above baseline in group AB from 45 to 405 minutes after treatment (Figure 1B). During the same period TTs in group AB were significantly increased compared to group S and group ACE. Thermal thresholds were not different between N and W. Analysis based on standardized thermal excursion (TE %) did not change the results (Figure 2A,B).

### Reaction to stimulation, sedation and side effects

The most frequent reaction to thermal stimulation at N was head shaking (54.2%) followed by rubbing the face (44.4%). Stimulation at W was mostly answered with skin twitching (86.7%) rarely with shaking the whole body (12.8%) or turning the head towards the stimulus (0.5%).

All horses became mildly to moderately sedated (score: 4 [1,9]) 15 minutes after ACE lasting for 60 to 150 minutes (99 ± 28 min). In group AB, a median sedation score of 3 [0,9] was reached and sedation lasted 30 to 210 minutes (85 ± 64 min). In this group 1 horse did not become sedated and another horse was deeply sedated (score: 8).

Gastrointestinal sounds were reduced or absent from 15 to 75 minutes after AB administration and lasted for 180 to 510 minutes (297 ± 124 min). The horse not showing signs of sedation in group AB developed signs of colic one hour after the experiment was completed. An impaction of the large colon was diagnosed and treated medically without further complications. In group AB, excitatory phenomena like restlessness and box walking were observed in 10 of 11 horses starting with waning sedative effects of acepromazine (Additional file 1). After treatment with AB restlessness persisted for 60 to 480 minutes (297 ± 160 min), in 7 horses restlessness started 15 to 75 minutes after injection, in 3 horses 165 to 225 minutes after drug administration. Increased salivation or tear production was observed in four horses 15 minutes after injection of AB for up to 60 minutes.

### Skin lesions

Mild swelling without pain on palpation occurred at the nostrils in group AB occasionally when the thermode was heated up to cut-out temperature. There were no skin lesions after administration of S or ACE.

### Buprenorphine pharmacokinetics

**Figure 3:**
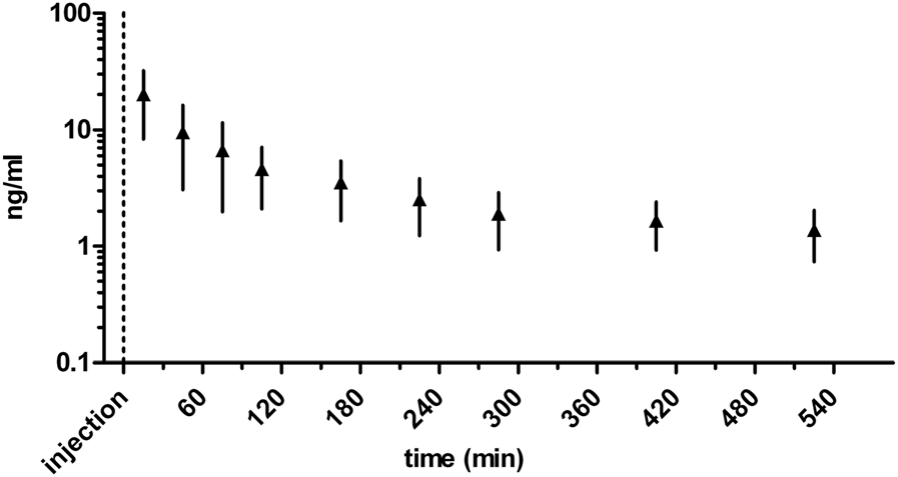
**Semilogarithmic buprenorphine serum concentrations.** Semilogarithmic buprenorphine serum concentrations (mean ± SD) over time in ten horses after intravenous injection of 0.0075 mg/kg buprenorphine.

The semilogarithmic serum concentration vs. time curve for buprenorphine in horses following IV administration is represented in Figure 3. Buprenorphine was detected for 8.75 hours in 10/10 horses. Harmonic mean of elimination half-life (*T*_1/2λ_) was 6.4 hours (Table 2). Mean serum concentration of buprenorphine at the last time point with increased TTs (405 minutes after AB) was 1.7 ± 0.7 ng/ml.

**Table 2:** **Pharmacokinetic parameters of buprenorphine after 0**.**0075 mg**/**kg bwt intravenously in ten horses**

**Horse**	**λ**_**z **_**(h**^**-1**^**)**	***T***_**1/2λ **_**(h)**	***V***_**d **_**(L/kg)**	**Cl (mL/min/kg)**	**AUC**_**0-∞ **_**(h·ng/mL)**	**MRT (h)**
1	0.12	7.02	3.89	6.40	19.54	6.98
2	0.06	12.09	1.88	1.80	69.39	12.48
3	0.06	8.86	2.90	3.78	33.12	9.25
4	0.12	5.59	0.83	1.71	72.93	5.12
5	0.12	6.79	1.03	1.76	71.17	6.54
6	0.18	3.51	0.56	1.84	67.91	3.47
7	0.06	9.89	3.07	3.59	34.81	11.96
8	0.12	6.85	0.92	1.56	80.33	7.61
9	0.18	4.30	1.69	4.55	27.47	5.09
10	0.12	7.47	3.74	5.78	21.62	9.56
Mean ± SD	0.11 ± 0.04	6.4 ± 2.63^*^	2.05 ± 1.25	3.28 ± 1.82	49.83 ± 24.37	7.81 ± 2.98

λ_z_, slope of the terminal phase; *T*_1/2λ_, half-life of terminal phase; *V*_d(area)_, volume of distribution; *Cl*, Clearance; *AUC*_0-∞_, area under the concentration-time curve extrapolated to infinity; *MRT* mean residence time. ^*^Harmonic mean ± pseudo SD.

## Discussion

Thermal thresholds determined by ramped contact heat at the head or the body to assess cutaneous anti-nociception in horses were comparable. Heat stimulation at both body sites allowed to recognize a clear end point, which was repeatable over several hours and responded to neuroleptic sedation and neuroleptanalgesia in a similar way.

Type of end point and end point detection in response to thermal stimulation at different body parts as nostrils versus withers might influence the TT. With stimulation at the withers a skin flick was the most clear end point, whereas stimulation at the nostrils resulted in head shaking or rubbing the face against an object [14]. In contrast to the reflex pathway of the skin flick reflex, which is mediated by the spinothalamic tract [10], responses to stimulation at the head like head shaking involves the trigeminal nerve and individual conscious perception and reaction to the stimulus [14]. Head jerking as part of the nocifensive trigemino cervical reflex was not observed with heat stimulation in the current study. Skin twitch at the withers is considered to be a more clear end point than the hoof withdrawal reflex, when testing effects of opioids because they interfere with the hoof withdrawal reflex as a result of the increase in spontaneous locomotor activity [5]. Opioids can also induce head nodding in horses and can make end point detection at the head more difficult. However, in the present study there was no difference in skin temperatures, threshold temperatures and changes in thresholds between stimulation at the head and the withers detected. A skin twitch is a very obvious and repeatable reaction, however, variability of thermal thresholds at the nostrils was in fact lower than at the withers, indicating that the defensive reaction at the head can repeatedly be detected despite being a more individual, behavioural response, which is not influenced by sedation.

Baseline thresholds were stable over 10 consecutive measurements and no conditioning to the stimulation procedure or the noxious stimulus was observed in our study. This is in agreement with previous studies in horses using a similar stimulation set up [9,14]. In contrast, horses became conditioned to the light stimulus with radiation heat stimulation [3] or to touch with mechanical pressure stimulation to the front leg before the stimulus became painful [15].

The standard heating rate of the thermode based system for cats (0.85°C/s) resulted in very variable threshold temperatures and skin lesions in horses [16]. Reducing the heating rates to 0.5°C/s and 0.2°C/s gave clearer end points and more consistent threshold temperatures but also caused restlessness during the lower heating rates [16]. In contrast, in pretrials with our horse population the very slow heating rates resulted in burns at the nostrils. In a previous study stimulation with heating rates of 0.6°C/s for the withers and 0.8°C/s for the nostril allowed a clear end point detection without inflammation or damage of deeper skin layers [14], but might have contributed to higher TTs compared to other studies and other species because of a delay in heat transfer to thermal nociceptors in the face of a rapid increase of temperature at the skin surface. The necessary differences in heating rates at the different stimulation sites to avoid skin damage might be related to differences in skin architecture and skin thickness resulting in differences in heat transfer [2]. The chosen heating rates are thought to activate C-fibre nociceptors and induce low thermal thresholds like it was shown in rats where slow rates of heating (0.6°C) evoked responses at low thresholds activating primarily C-nociceptors [17].

The mode of action of the used drugs is well known in horses and their sedative and analgesic actions were used to assess and compare the response of TTs at different body sites. The sedative effect of acepromazine was variable between horses with a maximum effect about 45 minutes after injection. The phenothiazine tranquilizer is widely used in equines [8,18] and is well known for its dose-dependent sedation [15,18] and lack of somatic antinociceptive effects [9,15]. Acepromazine has α_1_-adrenolytic activity, depresses the vasomotor center followed by hypotension, vasodilatation and increased digital blood flow [19-22]. These effects might interfere with the regulation of body and skin temperature. Rectal temperatures weren’t measured in this study, however, a late decrease in skin temperatures after ACE (Table 1) was observed at the withers. As contact heat thermal stimulation in the present study was performed during moderate ambient temperatures (14.8 ± 2.8°C) it can be assumed that the decrease in skin temperature was probably influenced by acepromazine.

As expected, buprenorphine, a semi-synthetic partial mu opioid agonist [23] increased thermal thresholds measured at the nostril as well as at the withers for several hours. In our study thermal thresholds were already increased above baseline at the first post treatment stimulation (15 minutes after AB). More frequent stimulation to detect earlier onset was not possible without active skin cooling. Cut-out was also reached 15 minutes after administration of butorphanol (0.1 mg/kg, IV) or buprenorphine (0.005 mg/kg, 0.0075 mg/kg, 0.01 mg/kg, IV) in a previous study in horses [9].

In the present study anti-nociceptive effects lasted for approximately 7 hours, which is in close agreement with a mean duration of anti-nociception of 7.8 hours after buprenorphine at the same dose reported in a previous study [9]. Mean serum concentration of buprenorphine at the last time point with increased TTs (405 minutes after AB) was 1.7 ± 0.7 ng/mL. Whereas levels of 0.59 ± 0.14 ng/mL (520 minutes after AB) did not result in analgesic effects anymore. For a more precise determination of minimal analgesic serum concentrations the threshold measurements would need to be performed more frequently. Therefore the 1.7 ± 0.7 ng/mL can only be considered a crude estimate for minimal analgesic serum concentrations of buprenorphine. Elimination half-life for buprenorphine was 6.4 hours in the current study, which might even be underestimated as serum concentrations did not fall below the limit of quantification in the terminal phase. However, the determined terminal half-life is comparable to 5.79 hours determined with a slightly lower dose of buprenorphine (0.006 mg/kg, IV) [24]. At a buprenorphine dose of 0.005 mg/kg buprenorphine IV elimination half-life was shorter with 3.58 hours [25] suggesting a dose dependent effect, differences in sensitivity of the buprenorphine analysis or an influence of acepromazine co-medication.

Almost all buprenorphine treated horses showed increased locomotor activity and signs of excitation for approximately 5 hours like it was described in previous studies [9,24-26]. Locomotor activity became evident when acepromazine effects declined, which might interfere with the detection of behavioural responses to thermal stimulation [3]. It is discussed controversially whether opioid induced spontaneous locomotor activity results from activation of the dopaminergic pathways [27,28] which might be ameliorated by acepromazine [20]. However, at the chosen doses the buprenorphine induced effects outlasted the sedative effects of acepromazine. In a previous study, sedative effects of acepromazine in horses were present for 240 minutes after IV injection of 0.15 mg/kg [20] a three times higher dose than in the current study. The degree of sedation after the acepromzine and buprenorphine combination was very variable and in some horses the duration of sedation seemed shorter than with acepromazine alone, which confirms the observation that sedative effects with a combination of buprenorphine and acepromazine in ponies were non-satisfying [29].

Another side effect of buprenorphine combined with acepromazine was reduction of gastrointestinal sounds for approximately 5 hours and abdominal discomfort in one horse 10 hours after drug administration. In other studies gastrointestinal borborygmi also decreased following buprenorphine which was attenuated when buprenorphine was given sublingually [9,24,26]. When hay was withdrawn 12 hours before intravenous buprenorphine, there were no signs of abdominal discomfort observed [9].

There was a slight increase in skin temperatures at both body sites after AB as long as measurements were performed at 30 minute intervals. Skin temperature in horses also rose after constant infusion rate with fentanyl [7] but an increase in skin temperature or body temperature after buprenorphine were not seen in previous studies [9,24]. It might be possible that the skin was warming up due to the more frequent heating cycles at the beginning of the observation period in the current study.

## Conclusion

Thermal contact heat stimulation at both described body sites (nostril and withers) give comparable results in the assessment of cutaneous nociception in horses. Buprenorphine combined with acepromazine results in several hours of cutaneous anti-nociception associated with locomotor stimulation and reduced gastrointestinal sounds/activity in healthy horses.

## Methods

### Animals

The study was approved by the Ethics Committee for Animal Experiments of Lower Saxony (33.12-42502-04-10/0136). Eleven warm-blood horses (4 geldings and 7 mares) weighing 600 +/− 95 kg, ranging from 5 to 23 years were used in this randomised, observer-blinded crossover study. All of them were determined to be healthy on the basis of results of a physical examination. Horses were free of acute and chronic lameness. All horses had the oral cavity and teeth examined and floated on a regular basis. The mares did not show signs of behavioural oestrus during the testing periods. During the study period the horses were housed in a box stall (4 × 4 m) and had free access to hay and fresh water.

### Experimental design

Each horse went through 6 different stimulation conditions including 2 different body sites and 3 different treatments. All horses in a familiar box stall without restraint during constant ambient temperatures (14.8 ± 2.8°C). At least 24 hours before starting the measurements the horses were allowed to get adapted to the environment (box stall) and the skin at the stimulation sites was shaved with a razor blade.

### Instrumentation

**Figure 4:**
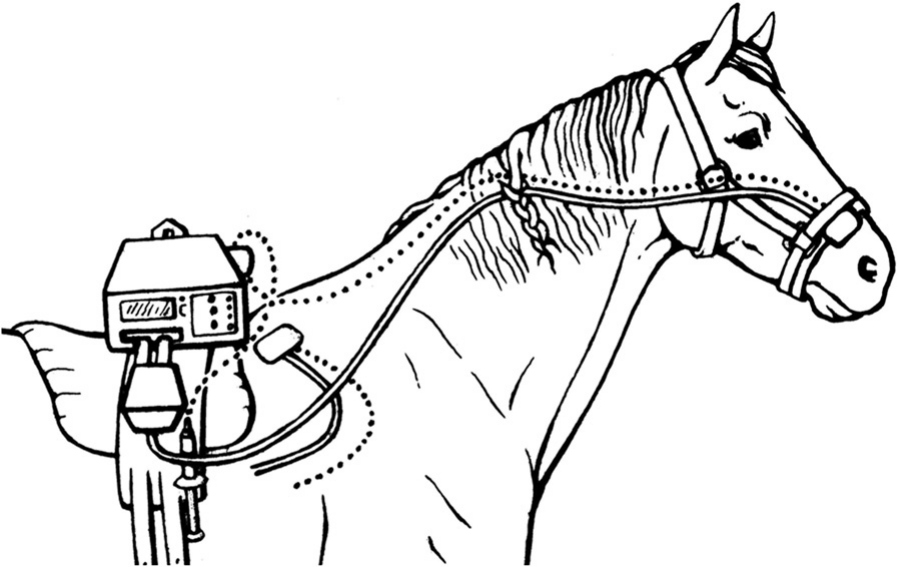
**Attachment of the Wireless Thermal Threshold testing system to the horse.** The thermal probes were placed at two different body parts of the horse (nostril, withers).

On the day of the experiment an intravenous catheter^a^ was aseptically placed in a jugular vein following subcutaneous administration of 1.5 ml 2% mepivacaine,^b^ secured with polyamide monofilament nylon suture.^c^ A wireless thermal threshold testing device^d^ was attached to the back of the horse with a belt and Velcro strips. The thermodes were placed at the withers and lateral to one nostril and kept at constant contact with the skin by means of an air bladder pressurized to approximately 80 mmHg (Figure 4).

### Experimental protocol

The thermal probes were allowed to equilibrate with skin temperature and the skin temperature was measured and recorded. The heating rate was set at 0.6°C/s for stimulation at the withers and 0.8°C/s for stimulation at the nostrils [16,30]. The cut-out temperature was set at 54°C for both locations. The heat controller was set to start randomly in order to produce a variable delay in the start of heating so that neither horse nor operator knew when heating began.

After complete instrumentation, a baseline measurement was performed by heating up the thermal probes fixed at the nostril and withers. Heating was stopped and the temperature recorded when the horse shook its head or rubbed the nose against an object or its legs, a skin twitch (reflex contraction of the cutaneous trunci muscle) occurred or the horse turned its head towards the stimulated site. The type of reaction to the thermal stimulus was documented. The position of the head, the ears and the nostril were recorded to assess whether the horse was nervous or distracted (head held high, ears in front position and flared nostrils). If the horse didn’t show a positive behavioural response to thermal stimulation, the cut-out temperature was recorded instead of the thermal threshold temperature. After each heating process, the probe was removed from the skin to allow cooling, and the probe was moved to a new area of skin for the next measurement.

Overall horses underwent nine thermal stimulations at each cutaneous site after treatment always in the same order, first at the nostril, five minutes later at the withers at regular intervals: 15, 45, 75, 105, 165, 225, 285, 405 and 525 minutes after drug injection.

For determination of the depth of sedation a score for position of the head as well as reactions to visual and acoustic stimulation was used (Table 3). Each parameter was classified from no sedation (0) to deep sedation (3). Visual stimulation was performed with a red fabric bag which was suddenly waved in front of the horse’s head. Acoustic stimulation was done by cracking a plastic bag behind the examiners back to avoid concurrent visual stimulation. Gastrointestinal sounds were recorded as normal, decreased or absent following auscultation in all 4 quadrants [25] and excitatory phenomena (restlessness, head shaking, increased tear production or salivation) were documented. When the experiment was finished, the skin was checked for lesions or swelling caused by the heating probe.

**Table 3:** Sedation score

**Head position**	0	lower lip at height of shoulder joint or higher
	1	lower lip between shoulder and olecranon
	2	lower lip between olecranon and carpal joint
	3	lower lip at carpal joint or lower
**Reaction to acoustic stimulation**	0	moving back, normal reaction
	1	mildly curbed reaction
	2	clearly curbed reaction
	3	no reaction
**Reaction to visual stimulation**	0	moving back, normal reaction
	1	mildly curbed reaction
	2	clearly curbed reaction
	3	no reaction
**Total score**	0	no sedation
	1-3	mild sedation
	4-6	moderate sedation
	7-9	deep sedation

### Treatments

Horses were randomly assigned to receive 1 out of 3 treatments, with a washout period of at least 14 days: saline solution^e^ IV (S); acepromazine^f^ 0.05 mg/kg IV (ACE); buprenorphine^g^ 0.0075 mg/kg combined with acepromazine 0.05 mg/kg IV (AB). Total volume of drug was made equal to 20 mL among different treatments and the drug was administered via a jugular catheter over 1 minute by a person not involved in the trial. When measurements were completed, the intravenous catheter was removed and iodine ointment^h^ placed over the puncture site.

### Sample collection and drug analysis

Blood samples were collected prior to drug administration, directly after baseline measurement of thermal stimulation (baseline), as well as after each thermal stimulation at 15, 45, 75, 105, 165, 225, 285, 405 and 525 minutes after drug administration. Ten ml of blood were collected from the jugular catheter into a syringe and discarded; then samples were collected (9 ml) and placed in test tubes^i^ with serum clot activator and were centrifuged two hours later with 3600 rotations/min (~2800 G) for 6 minutes. Then serum was transferred with disposable pipettes into three 1-ml cryogenic plastic storage tubes^j^ and stored at −80°C until analysis. The jugular catheter was flushed with 10 ml heparinzed saline solution before and after each blood sampling.

Serum samples of ten horses (samples from one horse got lost) were analysed with high-performance liquid chromatography with a tandem mass spectrometer (HPLC MSMS)^k^ in the commercial laboratory.^l^ Serum samples were thawed at room temperature. According to laboratory standard 5 ng Buprenorphine-D4 and 100 μl saturated Borax solution were added to the samples adjust to pH = 9.2. The mixture was extracted with 1 ml Ethyl acetate. After centrifugation the organic layer was separated and evaporated to dryness under nitrogen. The residue was dissolved with 100 μl acetonitrile and measured using an Agilent 1200SL HPLC system coupled to an Agilent 6460 mass spectrometer with an electro-spray ion source operated in positive mode. An Agilent Zorbax SB-C18 150 × 2.1 mm, 3.5 μm was used as stationary phase. Mobile phase was acetonitrile (A) and 0.1% formic acid in water (B). Gradient started at 95% B to 30% B within 7.5 min. Injection volume was 5 μl. Parameters of the Jet stream source: Drying gas Temp/Flow: 250°C/ 10 l min^-1^, Sheath gas 400/10, Capillary voltage 4500 V, Nozzel voltage 0 V. Calibration curves for buprenorphine were prepared by using buprenorphine spiked serum. Limit of quantitation was 0.05 ng/ml. The calibration curve was linear within 0.1 and 5 ng/mL. Intra-assay precision at buprenorphine concentrations of 10 ng/mL was 8.4%.

A protein precipitation technique using acetate was performed, followed by a solid phase extraction. The molecular mass of 427 Dalton corresponded to buprenorphine. Calibration curves for buprenorphine were prepared by using equine serum spiked with known buprenorphine concentrations. Limits of quantitation were determined to be 0.05 ng/mL. Intra-assay precision at buprenorphine concentrations of 10 ng/mL was 8.4%.

### Pharmacokinetic analysis

Serum concentrations of buprenorphine were analysed using commercial software.^m^ Noncompartmental analysis was used to derive the slope of the terminal phase (λ_z_), half-life of the terminal phase (*T*_1/2λ_), area under the concentration-time curve extrapolated to infinity (AUC_0-∞_) determined be the trapezoid method, apparent volume of distribution (*V*_d area_), and mean residence time (MRT) based on AUC _area_ according to standard pharmacokinetic calculations [31].

### Statistical and data analysis

Normal distribution of data was approved by visual assessment of the q-q-plots of the model residuals. Data were reported as mean ± standard deviation.

For standardizing thermal thresholds the following equation was used for calculating percent of thermal excursion:

TE  %   =  100 × ([T_T_ ‒ T_0_]/[T_c_ ‒ T_0_]). T_T_ is the thermal threshold temperature, T_0_ is the skin temperature and T_c_ is the thermal cut-out temperature [32].

Influence of drug administration and body site of the horse were analysed using a two-way analysis of variance (ANOVA) with repeated measurements within subjects and post-hoc Tukey-Kramer test for multiple pair wise comparisons. Statistical significance was attributed when *p* < 0.05. Analyses were carried out with commercial statistical software^n^ and graphs were prepared with graphical software.^o^

### Endnotes

^a^ 12 SWG, EquiCath^TM^ Fastflow, Braun Vet Care GmbH, Tuttlingen, Germany.

^b^ Scandicain^®^, AstraZeneca GmbH, Wedel, Germany.

^c^ Dafilon^®^ 1 metric, B. Braun, Aesculap AG, Tuttlingen, Germany.

^d^ Topcat Metrology Ltd., Little Downham, Ely, UK.

^e^ 0.9% natriumchlorid solution, B. Braun Melsungen AG, Melsungen, Germany.

^f^ Vetranquil^®^ 1%, Albrecht GmbH, Aulendorf, Germany.

^g^: Temgesic^®^, Essex Pharma GmbH, München, Germany.

^h^ Vet-Sept^®^, Albrecht GmbH, Aulendorf, Germany.

^i^ Vacuette^®^, Greiner Bio-One GmbH, Kremsmünster, Austria.

^j^ Eppendorf cuvettes, Eppendorf- Netheler- Hinz GmbH, Hamburg, Germany.

^k^ Agilent 6400 Sereis, Triple Quad LC/MS system, Santa Clara, CA.

^l^ LIPIDOMIX GmbH, Dr. rer. nat. M. Rothe, Berlin, Germany.

^m^ PK Solutions 2.0 ^TM^, Summit Research Services, Pharmacokinetics and Metabolism Software, Montrose, USA.

^n^ SAS^®^ version 9.2, SAS Institute, NC, USA.

^o^ GraphPad Prism^®^ version 5.03, GraphPad Software, Inc., CA, USA.

## Abbreviations

AB: Acepromazine and buprenorphine; ACE: Acepromazine; N: Nostril; S: Saline solution; TE %: Percentage of thermal excursion; TT: Thermal thresholds in °C; W: Withers; WTT2: Wireless thermal threshold testing device 2 (modified for horses).

## Competing interests

The authors declare that they have no competing interests.

## Authors’ contributions

SBRK conceived the project. CP accomplished the practical part of the study and was supported from KH and SBRK. KR carried out the statistical analysis. SBRK, KH and CP participated in interpretation of the study results. CP drafted the paper which was later revised by all co-authors through substantial contributions to the content of the paper. All authors read and approved the final manuscript.

## Supplementary Material

Additional file 1 **Side effect after AB administration.** The first horse seen in the video showed unconscious head nodding two minutes after AB administration for 3 minutes. One horse was deeply sedated (score: 8). Further horses were restless and showed box walking 3 – 4 hours after AB administration. Click here for file
